# Studies on the annealing and antibacterial properties of the silver-embedded aluminum/silica nanospheres

**DOI:** 10.1186/1556-276X-9-307

**Published:** 2014-06-17

**Authors:** Ko-Ying Pan, Chia-Hung Chien, Ying-Chih Pu, Chia-Ming Liu, Yung-Jung Hsu, Jien-Wei Yeh, Han C Shih

**Affiliations:** 1 Department of Materials Science and Engineering, National Tsing Hua University, Hsinchu 30013, Taiwan; 2 Department of Materials Science and Engineering, National Chiao Tung University, Hsinchu 30010, Taiwan; 3 Nano Technology Research Center, Industrial Technology Research Institute, Hsinchu 31040, Taiwan; 4 Department of Chemical and Materials Engineering, Institute of Nanomaterials, Chinese Culture University, Taipei 11114, Taiwan

**Keywords:** Silver-embedded aluminum/silica nanospheres, Sol-gel technique, Annealing, Chemical durability examination, Antibacterial test

## Abstract

Substantial silver-embedded aluminum/silica nanospheres with uniform diameter and morphology were successfully synthesized by sol-gel technique. After various annealing temperatures, the surface mechanisms of each sample were analyzed using scanning electron microscope, transmission electron microscope, and X-ray photoelectron spectroscopy. The chemical durability examinations and antibacterial tests of each sample were also carried out for the confirmation of its practical usage. Based on the result of the above analyses, the silver-embedded aluminum/silica nanospheres are eligible for fabricating antibacterial utensils.

## Background

During the last decades, owing to the greenhouse effect, the climatic anomaly has happened around our planet. The change in temperature is dramatic all over the world, the disease-causing germs threat humankind seriously, and consequently, various antibacterial materials, such as zeolite-based [1,2] and phosphate-based [3,4] compositions, ZnO nanoparticles [5,6], TiO_2_ nanoparticles [7-9], Ag nanoparticles [10-13], and core-shell silica-metal nanocompositions [14-16] are developed by researchers. Generally, the core-shell silica-silver nanocomposition is the most promising item among antibacterial materials due to its unique physical and chemical properties [17,18]. Nanosilver owns a large specific surface area and a high fraction of surface atoms; silica nanospheres, a typical dielectric core material for immobilized nanoparticles, has a good chemical and thermal stability, chemical inertness, large surface area, and outstanding compatibilities with several kinds of metal nanoshells. Moreover, sundry publication studies on core-shell silica-silver nanostructures focused on various manufacturing, optoelectronics, and catalytic and antibacterial features. It is common knowledge that the size, shape, and density of metal nanoshells and the combination of shells and cores mainly affect the presentations of the core-shell silica-silver nanocomposition, so many research groups have devoted their energies to discovering diverse ways for a very efficient sample. Therefore, throughout the previous literatures referring to the syntheses of the core-shell silica-silver nanocompositions, two approaches are majorly adopted: seed-mediated method and layer-by-layer (LbL) self-assembly technique [19,20].

The seed-mediated method [19], similar with electroless plating [21], includes two steps. In the first step, the surfaces of core materials are activated by chemicals or metals; in the second step, handling the redox reaction leads Ag^+^ ions into metallic Ag, which coats the SiO_2_ nanocores with tiny Ag nanoshells. In order to obtain the accurate thickness of shells, these two steps must be carried out again for several times, which means that this method is a great consumption and its products as impurities. The idea of LbL self-assembly technique is based on the alternate depositions derived from the electrostatic interactions between the charged components for multilayer growth on colloid particles. According the earlier published papers [22,23], LbL self-assembly technique is smart but time-consuming and unsuitable for production line. No doubt dealing with the drawbacks of above two major approaches would be the priority for researchers.

In this task, the authors synthesized the silver-embedded aluminum/silica nanospheres by sol-gel technique, a one-step method modified from Stöber method. The ideal silver-embedded aluminum/silica nanospheres for commercialization have to be manufactured under a high working temperature and keeping the color pure and white after work. For example, combinations of ceramics and antibacterial powders to make human-friendly utensils need to be heated and maintained at a high working temperature for calcination or sintering. However, there are few literatures putting emphasis on the annealing-induced properties of the silver-embedded aluminum/silica nanospheres, especially at high working temperatures above 600°C. Herein, authors did the annealing separately at 250°C, 400°C, 600°C, 800°C, and 1,000°C and investigate the annealing-induced properties of the silver-embedded aluminum/silica nanospheres via material and UV-visible (UV-vis) analyses. To follow the capabilities of these products realistically, the chemical durability test and antibacterial examination were handled as well.

## Methods

### Reagent

Five reagents are used in this task as follows: tetraethyl orthosilicate (TEOS, 98%, Alfa Aesar, Ward Hill, MA, USA), absolute ethanol (99.99%, Sigma-Aldrich Corporation, St. Louis, MO, USA), silver nitrate (99.9%, Mallinckrodt Pharmaceuticals, Dublin, Ireland), aqueous ammonia solution (30% to 33%, Sigma-Aldrich), and aluminum nitrate enneahydrate (Al(NO_3_)_3_ · 9H_2_O, Alfa Aesar). All reagents without additional purification and deionized (DI) water (Ω >10^18^) were used in all processes.

### Synthesis of silver embedded aluminum/silica nanospheres and annealing conditions

**Figure 1:**
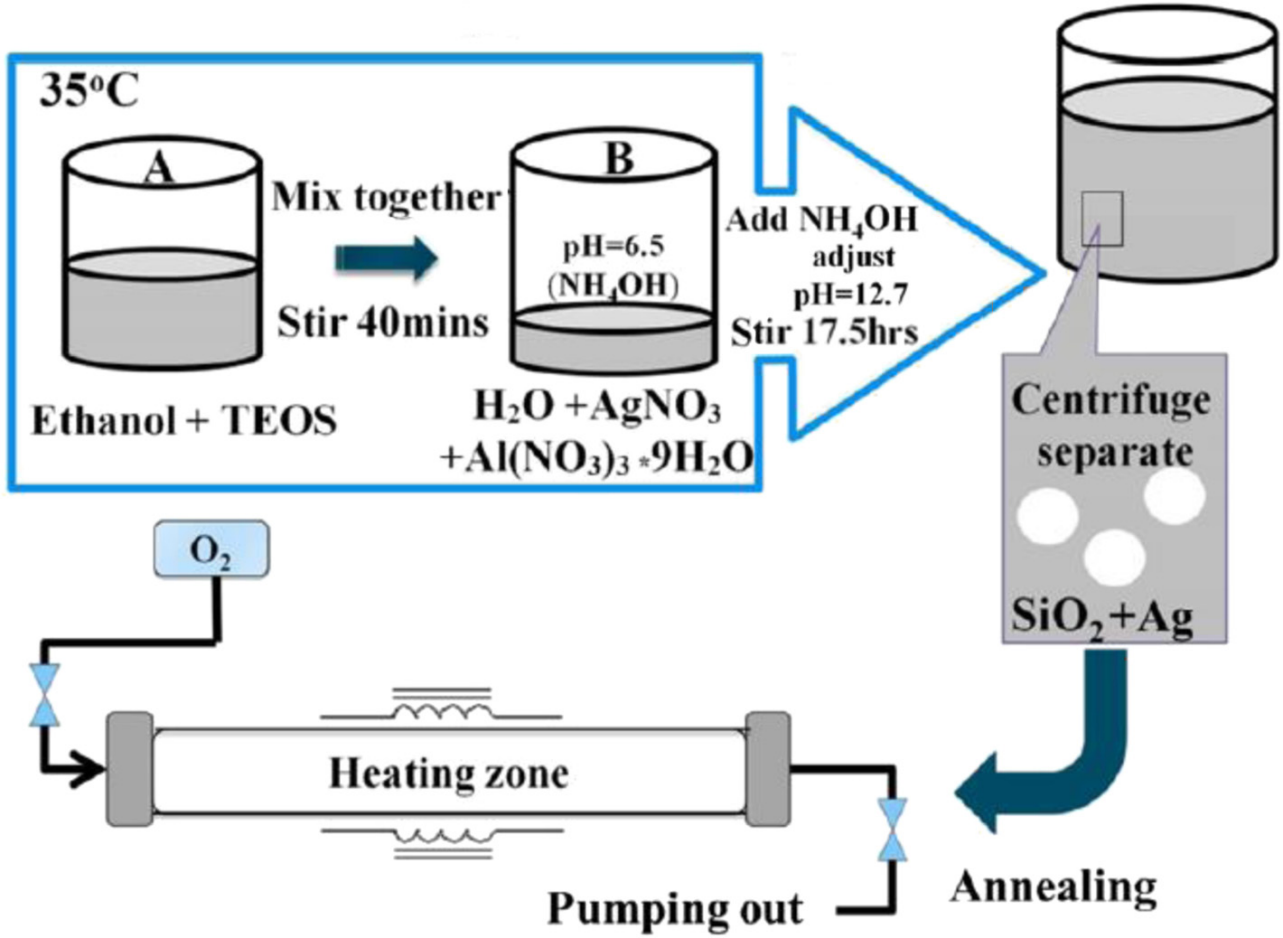
Fabrication of silver embedded on aluminum/silica nanospheres and annealing.

The silver-embedded aluminum/silica nanospheres, the as-prepared product, with diameter around 500 nm of silica particles and 7 nm of silver particles were prepared by sol-gel technique. Figure 1 is the schematic diagram of whole process flow used for silver-embedded aluminum/silica nanospheres, which includes the annealing process. In advance, two kinds of solutions, solutions A and B, had to be prepared. Solution A was prepared by mixing 4.2 g TEOS with 17.42 g ethanol by stirring for 10 min at 35°C. As to solution B, the mixture of 0.23 g aluminum nitrate enneahydrate, 0.1 g silver nitrate, and 4.38 g DI water was combined by stirring for 10 min at 35°C. Meanwhile, adding 0.5 ml of aqueous ammonia drop by drop into the solution B keeps the pH at approximately 6.5. Next, solutions A and B were mixed in a glass by stirring for 40 min at 35°C. Then, adding 100 ml aqueous ammonia into this combination maintains the pH at approximately 12.7 by stirring for 17.5 h at 35°C, thus saving the catalyze reaction time. While the above reaction was done, silver-embedded aluminum/silica nanospheres were collected by centrifugation. Eventually, the products were washed twice by ethanol and then dried in an oven at 50°C for 24 h.

After obtaining as-prepared powders, silver-embedded aluminum/silica nanospheres, the annealing processes were performed in the furnace. The as-prepared powders in an aluminum boat were placed in the heating area of the quartz tube, and the pressure inside the quartz tube was kept up at 5 × 10^−2^ Torr by a rotary pump. The working conditions of this process were as follows: (1) The temperature was raised separately to five working parameters (250°C, 400°C, 600°C, 800°C, and 1,000°C) with the heating rate of 1.7°C/min. (2) The temperature was maintained separately at five working parameters (250°C, 400°C, 600°C, 800°C, and 1,000°C) for 1 h. (3) Each sample was cooled separately to room temperature. In the above steps, (1), (2), and (3), O_2_ gas was continuously injected into the quartz tube at a constant flow rate of 4 sccm. The five annealing temperatures in this experiment were 250°C, 400°C, 600°C, 800°C, and 1,000°C, and these five samples obtained were termed as SAS-250, SAS-400, SAS-600, SAS-800, and SAS-1000, respectively.

### Characterizations of silver-embedded aluminum/silica nanospheres

#### Material analysis

The morphologies of each sample were characterized by scanning electron microscope (SEM, JSM-6500 F; JEOL Ltd., Tokyo, Japan) and high-resolution transmission electron microscope (HRTEM, JEM-2010). X-ray photoelectron spectroscopy (XPS) spectra were analyzed at an angle of 0° using PerkinElmer model PHI 1600 system (PerkinElmer Inc., Waltham, MA, USA) with Mg Kα line as an X-ray source and the energy resolution was 1.6 eV; all the deconvolutions of XPS curves were performed with the XPS Peak Fitting Program (XPSPEAK41, Chemistry, CUHK; Informer Technologies Inc., Copthall Roseau Valley, Dominica). UV-visible absorption spectra were recorded using a Hitachi U-3010 spectrophotometer (Hitachi Technologies, Shanghai, China).

#### Chemical durability test of silver-embedded aluminum/silica nanospheres in water

In this test, the focus was on the products after annealing at high temperature, so SAS-250 was ignored. First, each of the five samples of 0.25 g powder was dispersed in 10 ml water, and the Si, Al, and Ag ions released were determined after 2, 4, 6, 8, and 10 days of immersion. Second, 2 ml HNO_3_ (0.5 mM) was added into the retrieved solution. The concentrations of Si, Al, and Ag ions released from the sample into the water were measured using inductively couple plasma atomic emission spectroscopy (ICP-AES, ICAP 9000; Thermo Jarrell-Ash, Franklin, MA, USA) with a detectable limitation of 1 ppb.

#### Antibacterial examination of silver-embedded aluminum/silica nanospheres

Owing to the tracing of the antibacterial activity of products accepting annealing at a high temperature, SAS-250 was neglected in the above test. This antibacterial examination was based on Japanese Industrial Standards (JIS) Z 2801 method designed to test the antimicrobial activity or efficacy of plastics. Two bacterial types, *Escherichia coli* (*E. coli*) 8739 and *Staphylococcus aureus* (*S. aureus*) 6538P, were used for the antibacterial assays; *E. coli* and *S. aureus* belong to *Gram-negative* and *Gram-positive* bacteria, respectively. Thus, our products were verified as available for practical application in various antibacterial activities by this examination. As to the sample preparations of this test, first, a bulk using rolling depression was made by mixing 0.01 g sample powders and 0.99 g polyethylene (PE) powders at 180°C for 5 min. Next, transforming the bulk to plenty of crushed grains was done by a cutting machine. Then, forming the moderately crushed grains into an antibacterial film was carried out by depression machine at 180°C, and the average thickness of each film was less than 0.05 mm. To investigate the comparisons objectively, the placebo film was made of 100% PE as well.

The inoculum was dispersed and diluted with 1/500 NB as appropriate so that the amount of bacteria of the test inoculum was in the range of 2.5 × 10^5^ to 10 × 10^5^ cells/ml. After each test, the film was placed in a sterilized petri dish, using a pipette, and we took exactly 0.4 ml of the test inoculum and then instilled it onto each test film. Next, in order to incubate bacteria, the authors set the temperature of the petri dish containing the test film inculcated with the test inoculum at 35°C and a relative humidity of not less than 90% for 24 h. After incubation, the number of colonies was counted in a serially diluted petri dish. The results of this antibacterial examination will be discussed in ‘Antibacterial analysis of silver-embedded on aluminum/silica nanospheres’ section.

## Results and discussion

### Morphologies of silver-embedded aluminum/silica nanospheres

**Figure 2:**
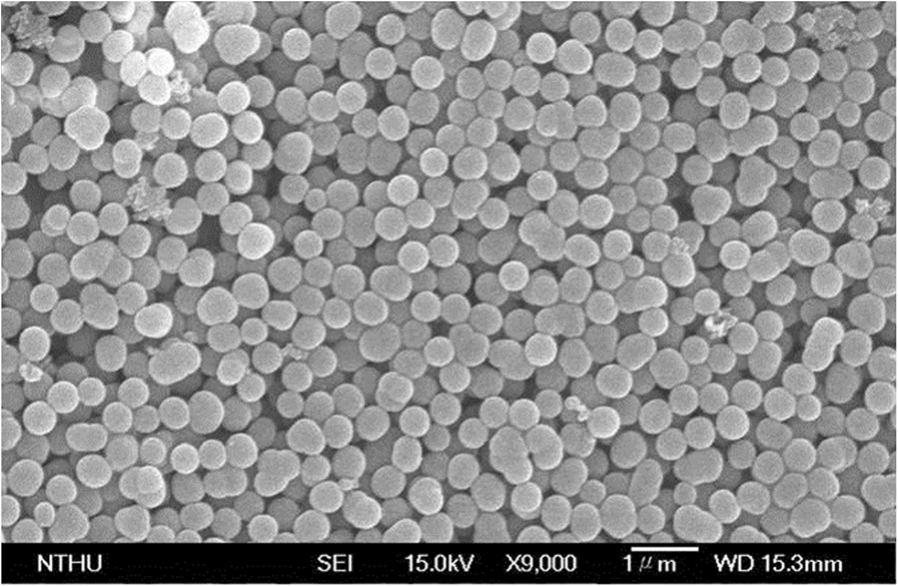
SEM image of silver-embedded silica nanospheres.

**Figure 3:**
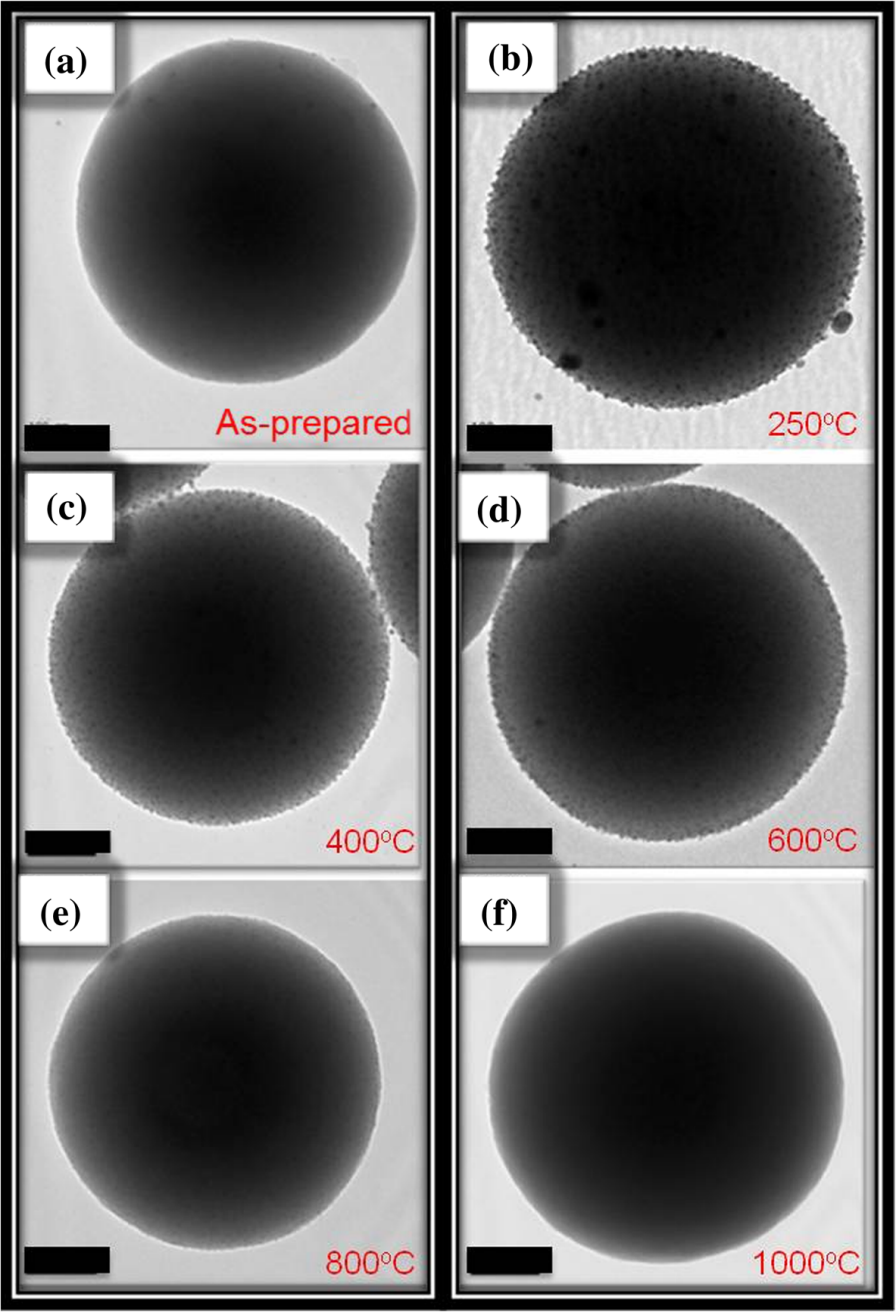
**TEM images. (a)** As-prepared sample. **(b)** SAS-250. **(c)** SAS-400. **(d)** SAS-600. **(e)** SAS-800. **(f)** SAS-1000. All scale bars are 100 nm.

Figure 2 shows the SEM image of abundant silver-embedded aluminum/silica nanospheres, the as-prepared sample by sol-gel method, with diameter of 500 nm [24]; these nanospheres are roughly equal in size. To observe the exact surfaces of each sample's single particle, the TEM images of each sample were taken in Figure 3. The reaction mechanisms of this sol-gel process can be explained as follows [25]. From ‘Synthesis of silver-embedded on aluminum/silica nanospheres and annealing conditions’ section and in Figure 1, two reactions in solution B are triggered by adding moderate ammonia solution. The first reaction includes two chemical processes, chemical equations 1 and 2, which are considered as follows:

(1) $${\mathrm{AgNO}}_{3}+{\mathrm{NH}}_{3}\cdot H_{2}O\to \mathrm{AgOH}↓+{\mathrm{NH}}_{4}NO_{3}$$

(2) $$Al^{3+}+3NH_{3}\cdot H_{2}O\to \mathrm{Al}{\left(\mathrm{OH}\right)}_{3}↓+3N{H_{4}}^{+}$$

Al ions and Ag ions are released by the hydrolysis of silver nitrate and aluminum nitrate enneahydrate in solution B. After adding moderate ammonia drops, the combination of hydroxyl ions and a lot of cations is reacted in solution B, thus producing AgOH and Al(OH)_3_.

The second reaction is the steps after chemical equation 1, which includes chemical equations 3 and 4.

(3) $$2\mathrm{AgOH}\to Ag_{2}O↓+H_{2}O$$

(4) $$\begin{array}{l} Ag_{2}O↓+H_{2}O+4NH_{3}\cdot H_{2}O\to 2\left[\mathrm{Ag}{\left(NH_{3}\right)}_{2}\right]\\ \mathrm{OH}+4H_{2}O \end{array}$$

**Figure 4:**
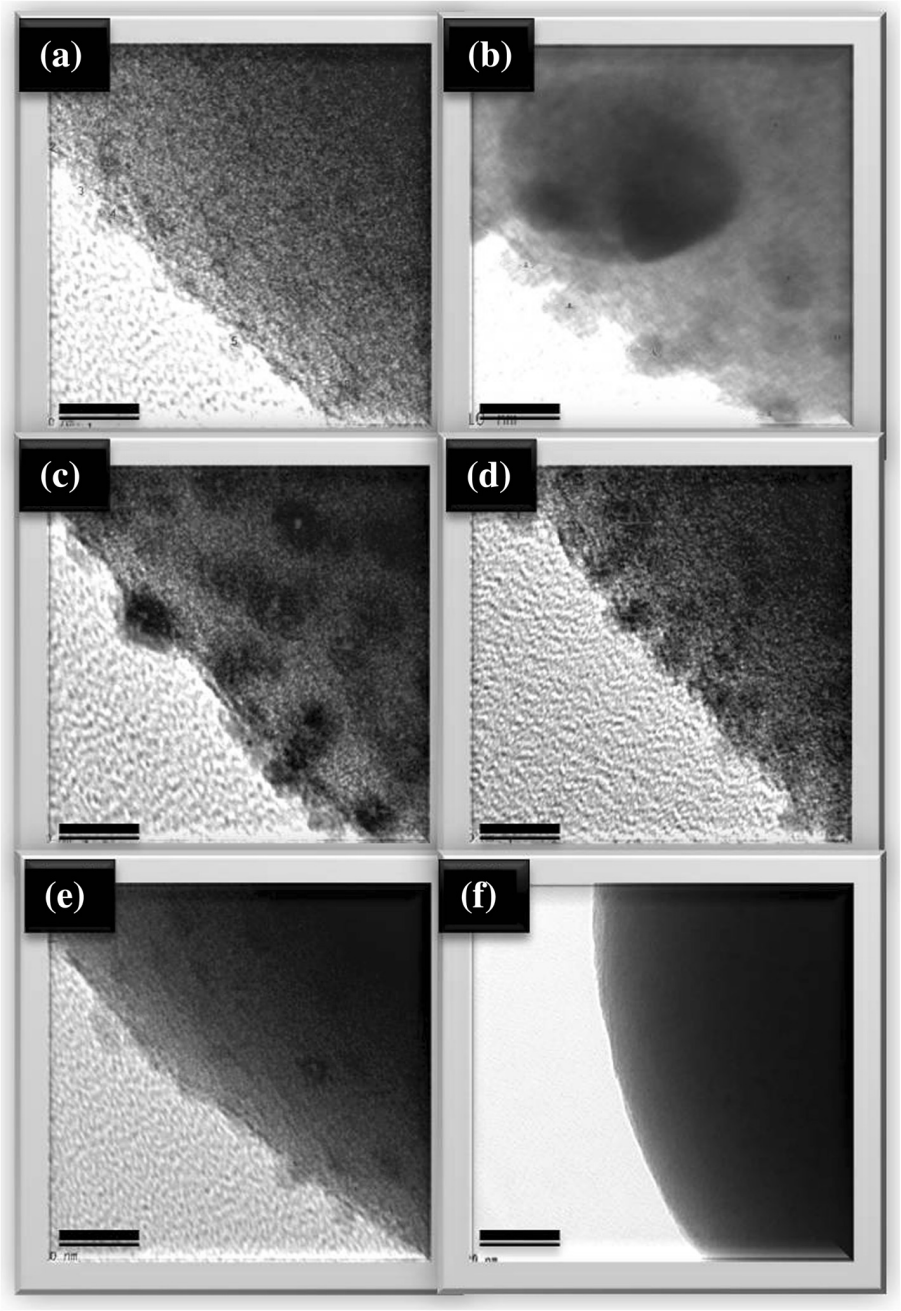
**Enlarged TEM images. (a)** As-prepared sample. **(b)** SAS-250. **(c)** SAS-400. **(d)** SAS-600. **(e)** SAS-800. **(f)** SAS-1000. All scale bars are 20 nm.

After getting AgOH, due to unstable state itself, AgOH becomes Ag_2_O spontaneously, as written in Equation 3. Meanwhile, as described in Equation 4, the ammonia still further reacts with Ag_2_O, hence, silver ammonia complex ions [Ag(NH_3_)_2_] dissolve in the solution B, which is the main source for Ag particles and Ag ions deposited on the surfaces of aluminum/silica nanospheres.To compare the detailed morphologies of each sample, Figure 3 was enlarged in Figure 4. From Figures 3a and 4a, the silver particles of the as-prepared sample are evenly distributed around the surface and the average size is about 2.87 nm. The silver particles of SAS-250 become large as well as rough, and the average size is about 6.56 nm, as shown in Figure 4b. Few silver clusters whose diameter is about 20 nm are also observed around the surface of SAS-250 in Figures 3b and 4b. The silver particle size of SAS-400 is smaller than that of SAS-250 and approximates to 5 nm; these silver particles are equally distributed around the surface, as shown in Figures 3c and 4c. The distribution of silver particles on SAS-600 is almost the same as it of SAS-400, and the dimension of silver particles of SAS-600 approximates 4 nm, smaller than it of SAS-400, as shown in Figures 3d and 4d. Judging from Figure 4e,d, it is clear that the amount of silver particles of SAS-800 is less than it of SAS-600. The silver particles of SAS-1000 disappeared around the surface, and the surface becomes very smooth, as shown in Figures 3f and 4f.

In terms of SAS-250, as shown in Figures 3b and 4b, the silver particles cluster becomes large, which can be accounted for as follows. Referring with the previous paper [26], after annealing from 200°C to 700°C, the oxygen atoms consist of two sorts, Si-OH (non-bridging oxygen) and Si-O-Si (bridging oxygen), in silver nanocrystals-doped silica films. The non-bridging oxygen acts as a key factor to undergo the cation exchanging process, as depicted in reaction (5):

(5) $$\mathrm{Si}-\mathrm{OH}+Ag^{+}\to \mathrm{Si}-O-\mathrm{Ag}+H^{+}$$

Therefore, due to this redox, the silver particles are deposited on the silica surface. Additionally, De et al. [26] indicate that the Ag characteristic peaks were exhibited in X-ray diffraction (XRD) pattern, since the sample accepted the annealing at and above 200°C. As for the sample which accepted the annealing under 200°C, the XRD pattern without Ag signal was also verified.

### XPS analysis of silver-embedded aluminum/silica nanospheres

**Figure 5:**
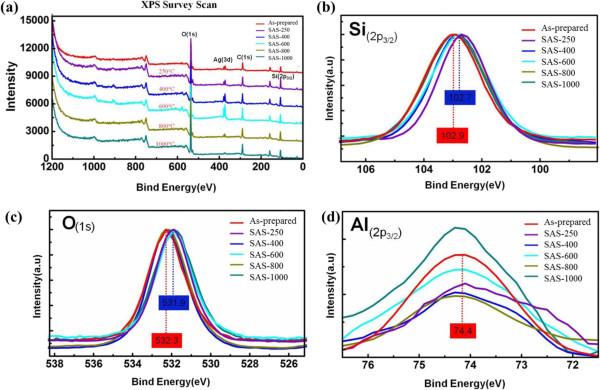
**XP spectra of silver-embedded silica nanospheres. (a)** Survey scan, **(b)** Si 2*p*, **(c)** O 1*s*, and **(d)** Al 2*p*.

**Figure 6:**
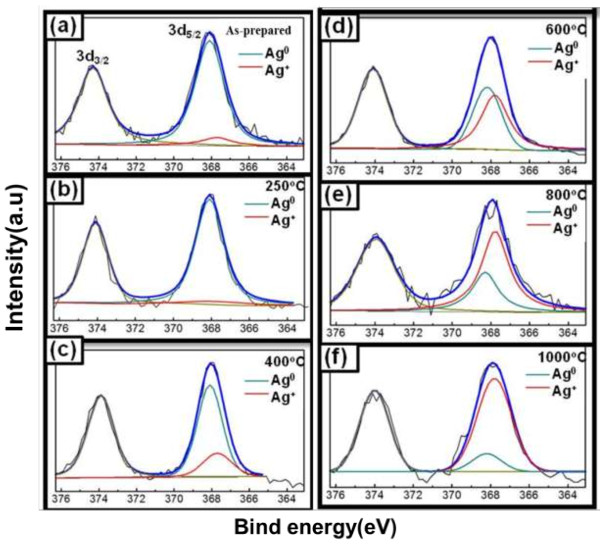
**XP spectra of Ag 3*d***_**3/2 **_**and 3*d***_**5/2 **_**in silver-embedded silica nanospheres. (a)** As-prepared, **(b)** SAS-250, **(c)** SAS-400, **(d)** SAS-600, **(e)** SAS-800, and **(f)** SAS-1000.

**Figure 7:**
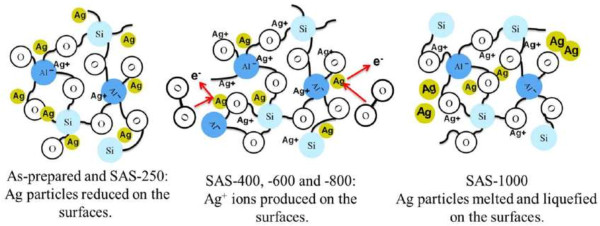
Mechanisms of silver-embedded aluminum/silica nanospheres at different annealing temperatures.

All binding energies were referenced to the C (1*s*) at 284.5 eV from carbon, and the spectrum deconvolutions were carried out with the XPS Peak Fitting Program. Figure 5a reveals the XPS survey scan of each sample. Figure 5b shows that the binding energies of Si (2*p*_3/2_) are 102.7 eV for SAS-400 and SAS-600, and 102.9 eV for as-prepared sample, SAS-800, and SAS-1,000. Figure 5c shows that the binding energies of O (1*s*) are 532.3 eV for the as-prepared sample and 531.9 eV for the other samples. Figure 5d shows that all samples' binding energy of Al (2*p*_3/2_) is 74.4 eV. Indeed, the matrix of all kinds of silver-embedded aluminum/silica nanospheres is stable silica nanospheres, which refers to the earlier paper [27]. Figure 6 shows that the binding energies of Ag (3*d*_5/2_) and Ag (3*d*_3/2_) are 368.1 eV and 374.4 eV, respectively. After deconvolutions of each sample's Ag (3*d*_5/2_) curves and integration of the areas under separated curves, the ratios of Ag^0^ to Ag^+^ can be obtained in Table 1. Ag^0^ and Ag^+^ represent the silver atomic state and silver ionic state, respectively. The ratios of Ag^0^ to Ag^+^ from as-prepared sample to ASA-1000 have decreased dramatically, which corresponds closely with the TEM images. These phenomena will be discussed as follows and cooperated in Figure 7. From the left part in Figure 7, in the as-prepared sample, the binding energy of Al-O (511 KJ/mol) is higher than that of Si-O (452 KJ/mol), so one aluminum atom substitutes for one silica atom to constitute a tetrahedral [AlO_4_]^−^ in silica cells. Because of the interaction between the [AlO_4_]^−^ structure and Ag^+^ ions, the Ag^+^ ions and the Ag particles exist in the surface of aluminum/silica nanospheres. When the annealing is handled at 250°C, the Ag^+^ ion percentage goes up due to the increased Ag^+^ ions from the inside of Ag-embedded aluminum/silica nanospheres induced by thermal energy. From Table 1 and the middle of Figure 7, in the annealing temperature ranging from 400°C to 800°C, the Ag^+^ ion percentages of SAS-400, SAS-600, and SAS-800 increased dramatically, which is being explained via two mechanisms as follows: (1) The Ag^+^ ions derive from inside to the surface in the Ag-embedded aluminum/silica nanospheres under an annealing at 400°C to 800°C. The thermal energy is not enough to provide these Ag^+^ ions on the surface for reductions of Ag atoms, thus increasing the percentage of Ag^+^ ions on the surface. (2) When annealing temperature is at 400°C to 800°C, the species of oxygen molecules become active, easily catching free electrons from Ag particles, and then the combinations of [AlO_4_]^−^ structures and Ag^+^ ions occur on the surfaces, thus raising the percentage of Ag^+^ ions. From Table 1 and the right of Figure 7, the percentage of Ag^+^ ions is the highest of all samples, when annealing is done at 1,000°C. Referring with the previous literature [28], in this range (annealing temperature above 800°C), the melting and liquefying during the annealing process of Ag particles on the surface is the root cause, hence the percentage of Ag atoms is much lower than the other samples, thus soaring the percentage of Ag^+^ ions on the surface.

**Table 1:** **XPS results of Ag**^**0 **^**and Ag**^**+ **^**by deconvolution of Ag (3** ***d***_**5/2**_**) curve**

	**Percentage of Ag**^**0**^	**Percentage of Ag**^**+**^	**Ratio (Ag**^**0**^**/Ag**^**+**^**)**
As-prepared	92.4	7.6	12.16
SAS-250	89.6	10.4	8.62
SAS-400	75.3	24.7	3.05
SAS-600	48.2	51.8	0.93
SAS-800	31.3	68.7	0.46
SAS-1000	14.1	85.9	0.16

In terms of silver oxides, no doubt they are easily decomposed under elevated temperature. However, there are no silver oxides in each sample of our experiments. The root cause is that the nanosized Ag would be produced during annealing and further vaporized at higher temperature with constant air flow.

### UV-visible spectrum analysis of silver-embedded aluminum/silica nanospheres

**Figure 8:**
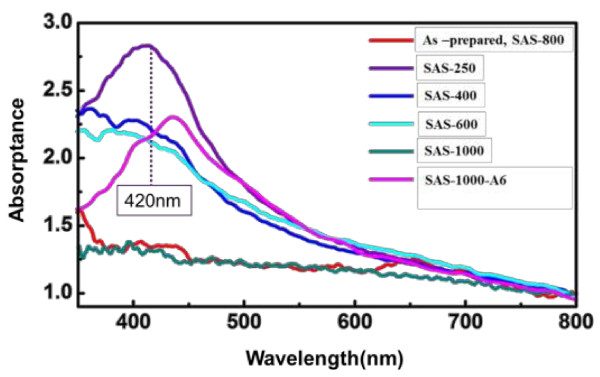
UV-visible spectra of silver-embedded silica nanospheres.

The UV-visible absorption spectra of the seven kinds of silver-embedded aluminum/silica nanospheres are illustrated in Figure 8, which are consistent with the surface morphologies of each sample. In principle, these UV-vis spectra appear in the interaction between absorption signals from silver nanoparticles and scattering signal signals from silica nanospheres [29]. When the size of silver nanoparticles is less than 5 nm, the surface plasmon resonance (SPR) absorption peak is not obviously shown at 420 nm in the wavelength scale [17,29]. The UV-visible spectra of as-prepared sample and SAS-800 show the same curve (red curve) due to their similar morphologies on their own surfaces. Owing to the densest and largest silver nanoparticles on the surface among all samples, the UV-visible spectrum of SAS-250 (purple curve) reveals the noticeable peak centered around 420 nm. Because the dimensions of silver particles are ranging from 3 to 5 nm and the scattering effects are derived from aluminum/silica matrixes, UV-visible spectra of SAS-400 (blue curve) and SAS-600 (sky blue curve) disclose the close resemblance tendency that their absorption peaks become broader and lower by comparing with the result of SAS-250. As to the UV-visible spectrum of SAS-1000 (deep green curve), due to the sample's surface without any nanoparticles, its signal is only derived from the scattering caused by aluminum/silica nanospheres, which looks like a line. By the way, the authors took SAS-1000 for further annealing at 600°C for 1 h under the atmosphere of 90% N_2_ + 10% H_2_ in the quartz tube, and this sample is termed SAS-1000-A6. The pink line is the UV-vis spectrum of SAS-1000-A6, and the red shift is found at the wavelength of 440 nm by comparing with the spectrum of SAS-250 (purple line).

### Chemical durability analysis of silver-embedded aluminum/silica nanospheres

**Figure 9:**
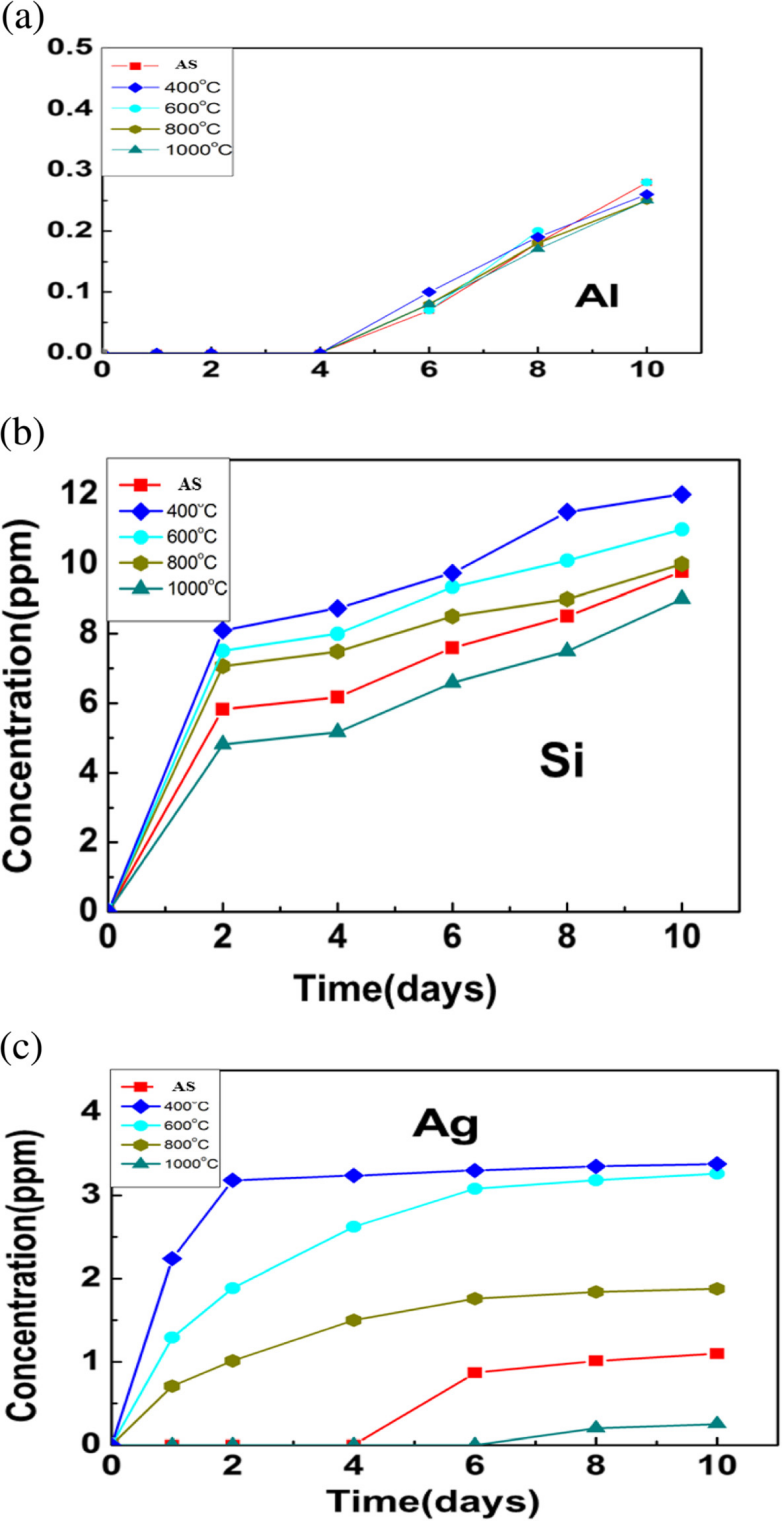
**Al, Si, and Ag ions released rates of silver-embedded silica nanospheres in water. (a)** Al ions released rate of silver-embedded silica nanospheres in water. **(b)** Si ions released rate of silver-embedded silica nanospheres in water. **(c)** Ag ions released rate of silver-embedded silica nanospheres in water.

Each sample's Al, Si, and Ag ions release tests in the water are determined, as sketched in Figure 9. The concentrations of the three types of ions in the water (*Y* axis) versus examination time (*X* axis) are recorded for each sample. From Figure 9a, the release of Al ions of each sample starts on the 4th day and the concentrations in the water are about 0.2 ppm on the 10th day, which denotes that chemical durability of Ag ions in each sample accords with the demands in practice. Because the silica content is the largest amount of all elements in each sample, its contact with water is more serious than Al and Ag elements. Therefore, the release of Si ions has begun since the test was starting, as shown in Figure 9b. Theoretically, the release of Ag ions in the water must be lower than 5 ppm, which being used in the practical. In other words, the samples in this research could be useful in reality. From Figure 9c, the release concentration of Ag ions of SAS-400 on the 10th is 3.2 ppm, which is the highest degree among all samples; the release concentration of Ag ions of SAS-1000 on the 10th day is 0.26 ppm, which is the lowest number among all samples and is in keeping with the morphologies on the surfaces of samples. On the surfaces of dense Ag particles, the amounts of Si-O-Ag bonds with polarity are larger than that of Ag-Ag bonds without polarity. Therefore, in water, the interaction between Si-O-Ag bonds and water is stronger than that between Ag-Ag bonds. Consequently, Ag ions from Si-O-Ag bonds release into water are easier than that from Ag-Ag bonds.

### Antibacterial analysis of silver-embedded aluminum/silica nanospheres

**Figure 10:**
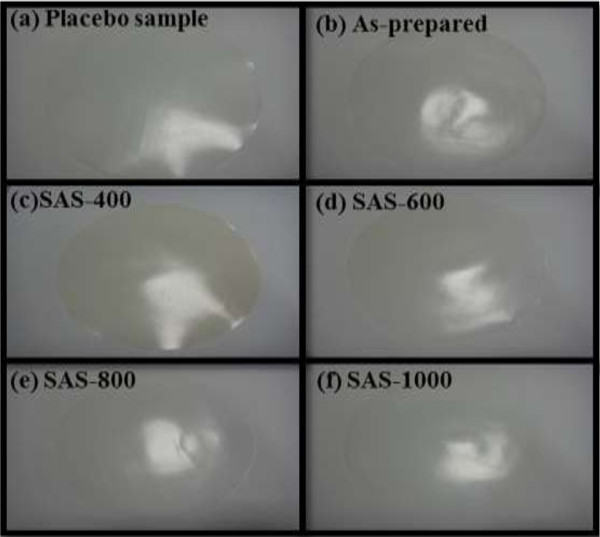
**Antibacterial examinations. (a)** Placebo film (100% PE). **(b)** Film of as-prepared sample. **(c)** Film of SAS-400. **(d)** Film of SAS-600, **(e)** Film of SAS-800. **(f)** Film of SAS-1000.

The petri dishes where the *E. coli* and *S. aureus* were cultivated on were contacted with each antibacterial film and placebo film, as shown in Figure 10. After a 24-h inoculation, the antibacterial activity is

(6) R=logB−logC

where *B* is the amount of bacteria after a 24-h inoculation of the placebo film, and *C* is the amount of bacteria after a 24-h inoculation of the antibacterial film. According to JIS R 1702 [30], if antibacterial activity (*R*) is greater than 2, it indicates that the sample has antibacterial effects. The results of antibacterial examinations are listed in Table 2. It is self-evident that all samples are qualified but SAS-1000, which infers the surfaces of SAS-1000 without Ag ions adsorbed.

**Table 2:** Results of the antibacterial examinations

**Test items**		**Test results**						**Test methods**
		**Place-PE**	**As-prepared**	**SAS-400**	**SAS-600**	**SAS-800**	**SAS-1000**	
*Staphylococcus aureus* ATCC 6538P	*B*	2.8 × 10^5^	2.8 × 10^5^	2.8 × 10^5^	2.8 × 10^5^	2.8 × 10^5^	2.8 × 10^5^	JIS Z2801:2000/AMDI:2006
	*C*	*-*	<10	<10	<10	<10	2.9 × 10^4^	
	*R*	*-*	4.4	4.4	4.4	4.4	1	
*Escherichia coli* ATCC 8739	*B*	9.3 × 10^6^	9.3 × 10^6^	9.3 × 10^6^	9.3 × 10^6^	9.3 × 10^6^	9.3 × 10^6^	Inoculation time 24 h
	*C*	*-*	<10	<10	<10	<10	7.2 × 10^6^	
	*R*	*-*	6	6	6	6	0.1	

## Conclusions

In summary, sol-gel technique has been successfully expanded to synthesize the silver-embedded aluminum/silica nanospheres, and the diameter of the nanospheres is about 500 nm. The ratios of Ag^+^ ions to Ag atoms on the surface of each sample deeply affect the optical and chemical features and are proportional to the annealing temperatures. Judging by the chemical durability and antibacterial determinations for antibacterial usages in practice, all samples are eligible but SAS-1000, which indicates that silver-embedded aluminum/silica nanospheres would be the promising candidate for manufacturing antibacterial utensils.

## Competing interests

The authors declare that they have no competing interests.

## Authors’ contributions

KYP wrote the whole manuscript and carried out some experiments and the data analysis. CHC and YCP performed some experiments and data analysis. CML carried out the antibacterial examinations. YJH, JWY, and HCS provided the ideas, information, and facilities of this task. Mostly, HCS organized the final version of this paper. All authors read and approved the final manuscript.

## Authors’ information

KYP is a PhD student at the National Tsing Hua University in Taiwan, R.O.C. and have devoted much attention to the research of nanomaterials. CHC got a master's degree from the National Tsing Hua University and now is an engineer in TSMC. YCP got a PhD degree from National Chiao Tung University and is currently a postdoctoral fellow at UC Santa Cruz in USA. CML is currently a senior supervisor in the Nano Technology Research Center. YJH is currently a vice professor at National Chiao Tung University. JWY is currently a professor at National Tsing Hua University. HCS is currently a professor at National Tsing Hua University and Chinese Culture University.
